# Sexual rehabilitation across the cystectomy care pathway: pragmatic guidance for multidisciplinary teams; a narrative review^✰^

**DOI:** 10.1016/j.ijnsa.2026.100601

**Published:** 2026-06-16

**Authors:** Marek Broul, Aneta Hujova, Michaela Liegertova, Jaroslava Banyrova, Michal Vostry, Klara Dadova

**Affiliations:** a Department of Sexology, Krajska zdravotni a.s., Masaryk Hospital, Usti nad Labem, Czech Republic; b Department of Urology, Krajska zdravotni a.s., Litomerice Hospital, Litomerice, Czech Republic; c Faculty of Health Studies, Jan Evangelista Purkyne University, Usti nad Labem, Czech Republic; d Faculty of Science, Jan Evangelista Purkyne University, Usti nad Labem, Czech Republic; e Faculty of Physical Education and Sport, Charles University, Prague, Czech Republic; f Private Healthcare Facility, Jaroslava Banyrova, Usti nad Labem, Czech Republic

**Keywords:** Radical cystectomy, Sexual rehabilitation, Oncology nursing, Pelvic floor, Survivorship, Ostomy, Body image

## Abstract

**Background:**

Radical cystectomy with urinary diversion can substantially affect sexual function, body image, and intimate relationships. Structured sexual rehabilitation guidance that is specific to cystectomy and usable within nursing and rehabilitation pathways remains limited, especially for women and for patients living with urinary diversion.

**Objective:**

To develop a practice-oriented, evidence-informed and consensus-based framework for sexual rehabilitation across the cystectomy care pathway.

**Methods:**

We conducted a targeted narrative review of literature published between January 2000 and June 2025 in the Medical Literature Analysis and Retrieval System Online via PubMed, Embase, Cumulative Index to Nursing and Allied Health Literature, and the Cochrane Library, supplemented by cancer survivorship, ostomy, pelvic rehabilitation and sexual medicine guidance. Two reviewers screened records and mapped evidence by recovery phase, sex-related anatomy, diversion type, presenting problem and delivery setting. Recommendations were refined through two rounds of multidisciplinary author-panel consensus. The work was aligned with the Scale for the Assessment of Narrative Review Articles, intervention reporting principles from the Template for Intervention Description and Replication checklist, selected Appraisal of Guidelines for Research and Evaluation principles, and sex- and gender-sensitive reporting guidance.

**Results:**

The revised framework is organised into prehabilitation, early postoperative care and late rehabilitation. It distinguishes nurse-led, shared interdisciplinary and specialist-only actions; specifies transition criteria between phases; integrates diversion-specific intimacy planning; and provides safety precautions for pelvic floor, manual therapy, pharmacological and psychosexual interventions. Outcome monitoring uses the International Index of Erectile Function, 5-item version; the Female Sexual Function Index, 19-item version; and the Body Image Scale, supplemented by goal-based and process indicators.

**Conclusions:**

The framework offers a cautious, adaptable route for integrating sexual health assessment, education, pelvic rehabilitation, stoma-related support, psychosexual communication and referral into routine cystectomy survivorship care. It should be implemented with local scope-of-practice governance and evaluated prospectively, including patient-partner co-design and long-term outcomes beyond 12 months.

**What is already known**

• Radical cystectomy and urinary diversion commonly disrupt sexual function, body image and intimate relationships.
• Sexual health needs after cystectomy are often under-assessed and under-treated, particularly for women and for people living with urinary diversion.
• Existing guidance rarely combines oncology nursing, stoma care, pelvic rehabilitation and psychosexual referral into one implementable pathway.

Alt-text: Unlabelled box dummy alt text

**What this paper adds**

• This paper provides a three-phase sexual rehabilitation framework spanning prehabilitation, early postoperative recovery and late survivorship care.
• The framework separates nurse-led actions, shared interdisciplinary care and specialist referral-only components to support international scope-of-practice adaptation.
• The revision adds explicit evidence labels, phase-transition criteria, safety precautions, outcome measures, limited-resource options and a research agenda for prospective evaluation.

Alt-text: Unlabelled box dummy alt text

## 1 Introduction

Radical cystectomy with urinary diversion remains the standard treatment for muscle-invasive bladder cancer and selected high-risk non-muscle-invasive disease (van der Heijden et al., 2025). Beyond urinary reconstruction, radical cystectomy can affect sexual function, including erectile dysfunction, ejaculatory and orgasmic changes, climacturia, lubrication deficits, dyspareunia, altered genital sensation and orgasmic changes, with downstream impacts on body image, confidence, relationships and quality of life (Modh et al., 2014).

Unmet needs are not limited to physiology. Reviews and qualitative syntheses in ostomy and urinary diversion populations describe body-image disruption, avoidance of intimacy, partner concerns, fear of leakage or odour and reduced sexual activity (Donegan and Kingston, 2022; Taylan et al., 2025; Hedgepeth et al., 2010; Westerman et al., 2021). In bladder cancer specifically, prospective and cross-sectional work has linked urinary diversion with body-image and sexuality outcomes, although evidence remains heterogeneous and affected by patient selection, surgical technique and diversion type (Hedgepeth et al., 2010; Westerman et al., 2021).

Clinical practice also reflects major implementation gaps. Women report substantial unmet educational needs about sexual function after radical cystectomy, and providers report non-routine counselling, particularly for female patients, across domains such as baseline function, postoperative risk, nerve- or organ-sparing options, follow-up counselling and referral (Westerman et al., 2021; Gupta et al., 2020). Cancer survivorship guidance identifies sexuality as a core component of care and supports systematic assessment and intervention pathways (Sanft et al., 2024; Carter et al., 2018). However, cystectomy-specific guidance that can be used by oncology nurses, stoma or urostomy nurses and pelvic health physiotherapists remains limited.

Aim and target users: This paper provides a pragmatic but explicitly adaptable practice framework for sexual rehabilitation across the cystectomy pathway. It is primarily intended for pelvic health physiotherapists, oncology nurses and stoma or urostomy nurses working within multidisciplinary cystectomy services. The framework identifies which actions can usually be nurse-led, which require shared interdisciplinary delivery and which should be referred to urology, gynaecology or menopause care, psychology or psycho-oncology, sexology or sexual medicine.

The guidance is not presented as a definitive clinical guideline or as evidence of intervention effectiveness. It is a prototype practice framework intended to standardise assessment, communication, safety screening and referral while supporting local adaptation according to staffing, regulation, scope of practice and patient priorities.

## 2 Methods

Design and reporting orientation: We developed a practice-oriented narrative review with consensus-based recommendations. The review and reporting approach was aligned with the Scale for the Assessment of Narrative Review Articles to improve clarity of the aim, search description, referencing, scientific reasoning and evidence handling (Baethge et al., 2019). Because the output is an intervention-like practice framework, the description of the protocol was also checked against intervention reporting principles from the Template for Intervention Description and Replication checklist (Hoffmann et al., 2014). Selected principles from the Appraisal of Guidelines for Research and Evaluation instrument, particularly scope, stakeholder relevance, clarity of presentation, applicability and editorial independence, were used as quality prompts rather than as a formal guideline appraisal (Brouwers et al., 2010). Sex- and gender-sensitive reporting principles informed the discussion of sex-specific anatomy, gender-diverse care and limitations (Heidari et al., 2016).

Literature search: We searched the Medical Literature Analysis and Retrieval System Online via PubMed, Embase, the Cumulative Index to Nursing and Allied Health Literature, and the Cochrane Library for English and Czech literature from January 2000 to June 2025. Search concepts included radical cystectomy, urinary diversion, sexual function, sexual dysfunction, pelvic floor, dyspareunia, erectile dysfunction, ostomy or urostomy, body image, intimacy, quality of life, rehabilitation, physiotherapy, psychosexual therapy, oncology nursing and stoma nursing. We also screened relevant clinical guidance on cancer-related sexual problems, survivorship care, menopause care and parastomal hernia prevention (Sanft et al., 2024; Carter et al., 2018; Antoniou et al., 2018; The North American Menopause Society, 2020).

Eligibility and narrative synthesis: We included studies and reviews reporting sexual, body-image or intimacy outcomes after radical cystectomy, and evidence or guidance on pelvic floor rehabilitation, ostomy-related intimacy, cancer survivorship sexual health, menopause-related vulvovaginal symptoms, psychosexual intervention and implementation strategies relevant to cystectomy pathways. Two reviewers screened titles, abstracts and full texts; disagreements were resolved by discussion. Findings were extracted into a narrative matrix by recovery phase, sex-related anatomy, diversion type, presenting concern, candidate intervention, safety issue, delivery discipline and referral trigger. The synthesis prioritised clinical usability and transparency about uncertainty rather than pooled effect estimation.

Quality appraisal and limits of inference: We did not conduct a formal risk-of-bias assessment because the evidence base combined observational cystectomy cohorts, qualitative studies, reviews, guidelines and extrapolated pelvic oncology or sexual medicine evidence, and because the framework does not make comparative effectiveness claims. Instead, each recommendation was assigned an evidence label and caution statement. This should be interpreted as implementation guidance for prospective testing, not as proof that a specific component improves sexual outcomes.

Consensus process: Draft recommendations were circulated to a six-member multidisciplinary author panel with expertise spanning urology and uro-oncology, sexology and sexual medicine, pelvic health physiotherapy and rehabilitation, behavioural health, health sciences and implementation-focused scholarship. Two structured rounds were conducted. Items were retained when at least 75% agreement was reached. Items with lower agreement were revised, reframed as options, moved to specialist referral pathways or accompanied by explicit caution statements. No item with unresolved safety concern was retained as routine practice. This was an author-group consensus process; no external patient, partner, frontline nursing or independent expert panel was convened, and external validation remains necessary before the framework is treated as authoritative guidance.

Panel and nursing positioning: The framework is intended for nursing and multidisciplinary pathways, but it should not be read as expanding nursing scope beyond local regulation, training and credentialing. Nursing-relevant actions are explicitly separated from pelvic health physiotherapy and specialist interventions. Services adopting the framework should review it with local oncology nursing, stoma nursing and clinical governance leads before implementation.

Ethics: This work synthesises published literature and expert consensus and did not involve recruitment, intervention delivery, patient-identifiable data or access to unpublished patient records. Ethical approval and informed consent were therefore not required. For studies involving human participants, we relied on the ethical governance reported by the original publications.

Evidence labels used throughout the framework are defined in Table 1.

**Table 1:** Evidence labels used for recommendations.

Label	Operational definition	How clinicians should use it
Cystectomy-specific evidence-supported	Recommendation informed directly by radical cystectomy or bladder cancer studies, including observational cohorts, trials, qualitative work or cystectomy-specific reviews.	Can be considered relevant to cystectomy care, while still accounting for study design, population and local context.
Extrapolated	Recommendation adapted from broader pelvic oncology, pelvic surgery, ostomy, menopause, sexual medicine, psychosexual or cancer survivorship evidence.	Use cautiously, explain uncertainty to patients and monitor outcomes.
Consensus good practice	Safety or implementation step judged clinically reasonable by the panel but with limited direct research evidence.	Use as a pragmatic starting point; adapt locally and evaluate prospectively.

### 2.1 Results: practice framework

Only a limited number of recommendations in this framework are directly derived from post-cystectomy studies. Most recommendations are extrapolated from related evidence or represent consensus good practice and should therefore be applied cautiously, adapted to local context and evaluated prospectively.

#### 2.1.1 Implementation overview and intended setting

The framework is designed for routine cystectomy pathways, including preoperative education, inpatient or pre-discharge teaching, outpatient pelvic rehabilitation, stoma follow-up and survivorship review. It can be implemented in centres without in-house sexology by using structured sexual health communication, clear referral routes and a minimum viable pathway that prioritises safety, permission-giving and referral initiation.

Minimum viable delivery is a consensus-based implementation proposal rather than an empirically validated dose: one prehabilitation session when feasible; one or two early postoperative contacts during the first 8 weeks; and three to six late rehabilitation contacts over 3 to 6 months for patients with ongoing goals or dysfunction. Services with limited capacity may begin with universal screening and education, a stoma-specific intimacy checklist, one follow-up prompt at 8 to 12 weeks and referral pathways for complex needs.

**Fig. 1:**
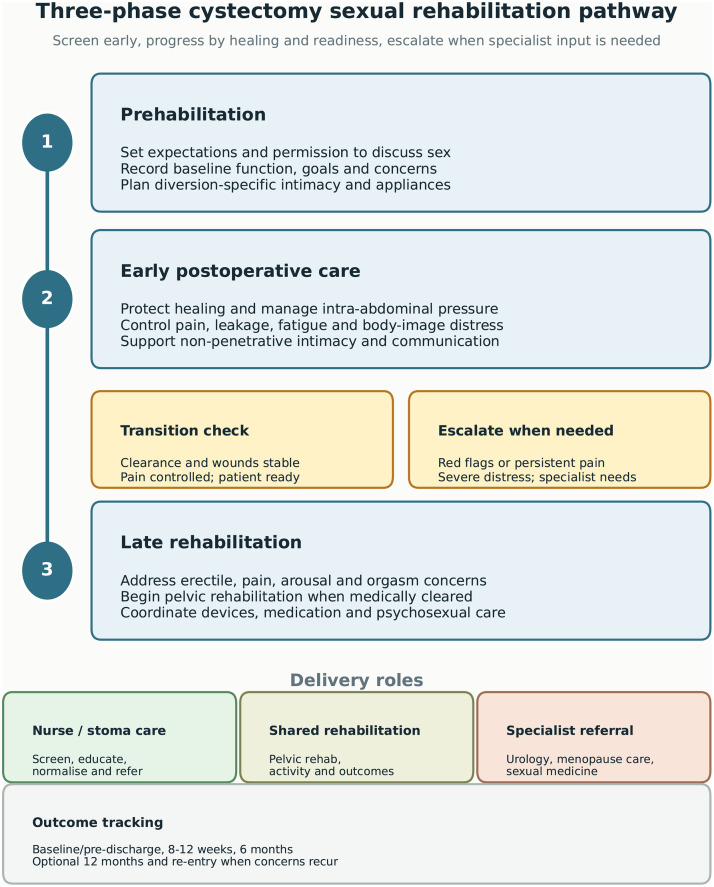
Three-phase cystectomy sexual rehabilitation pathway with transition checks and referral interfaces.

Fig. 1 summarises the phase-based pathway, transition checks and referral interfaces.

#### 2.1.2 Outcome measures and follow-up schedule

Outcome tracking should be simple enough for routine clinics. Baseline assessment is preferred preoperatively, or at pre-discharge if preoperative contact is not possible. Reassessment at 8 to 12 weeks supports transition into targeted late rehabilitation if healing permits. Six months is a pragmatic minimum reassessment point, with optional 12-month follow-up and re-entry because sexual recovery and body-image adjustment may continue beyond the first postoperative year.

We prioritised brief, validated measures directly linked to the domains targeted by the framework: the International Index of Erectile Function, 5-item version, for erectile function (Rosen et al., 1997, 1999, 2011); the Female Sexual Function Index, 19-item version, for female sexual function domains (Rosen et al., 2000; Wiegel et al., 2005); and the Body Image Scale for body image concerns in cancer populations (Hopwood et al., 2001; Shunmugasundaram et al., 2022). Broader quality-of-life tools such as the European Organisation for Research and Treatment of Cancer quality-of-life modules may be useful in research or comprehensive survivorship clinics, but were not selected as core tools because they add patient burden and are less specific to the rehabilitation targets of this framework.

Because cystectomy-specific benchmarks are not established, early implementation should focus on process indicators and local audit baselines. For example, services could set incremental targets based on local baselines, such as increasing documentation of sexual health screening and diversion-specific intimacy education from current levels toward 70% to 80% of eligible patients. These figures are author-panel consensus examples, not evidence-based thresholds or minimum institutional standards. Actioning of triggered referrals should be monitored within a locally defined time frame, and outcome indicators should be interpreted cautiously and in relation to patient-defined goals.

#### 2.1.3 Training and competency levels

Foundational competencies for all clinicians who initiate the pathway include permission-giving language, privacy and consent, awareness of common cystectomy-related sexual sequelae, recognition of stoma-related intimacy concerns, ability to screen for distress and red flags, and knowledge of local referral routes. Intermediate competencies, usually shared between oncology or stoma nursing and pelvic health physiotherapy, include diversion-specific intimacy planning, safe activity progression, body-image coaching and routine outcome tracking. Advanced competencies, such as internal pelvic floor examination, manual therapy, graded dilator programmes, complex sexual medicine decision-making and trauma-focused therapy, require discipline-specific training and credentialing.

#### 2.1.4 Phase transition criteria

Transition from prehabilitation to early postoperative care occurs at surgery or at the earliest postoperative teaching opportunity. Transition from early postoperative care to late rehabilitation should be based on surgical clearance, adequate wound and tissue healing, manageable pain and fatigue, no active infection or unresolved wound complication, stable diversion management, and patient readiness. Patient readiness is not a score-based requirement; it may include self-reported willingness to attempt non-penetrative intimate activity, absence of severe anxiety or avoidance primarily driven by fear, and confidence that the patient can pause or stop activity if symptoms occur. Penetrative intercourse, internal pelvic floor assessment, manual therapy, high-load core activity and device-based sexual rehabilitation should be deferred until these criteria are met. Escalation should use the same red flags listed in the phase sections, including fever, wound deterioration, new abdominal or stomal bulge, new prolapse symptoms, acute bleeding, escalating pelvic pain, uncontrolled leakage, suspected urinary diversion complication or severe psychological distress.

#### 2.1.5 Phase 1: prehabilitation

Objectives are to establish realistic expectations, identify sexual and relational goals, introduce body and pelvic awareness, invite partner involvement when the patient wants this, and make diversion-specific intimacy planning concrete. Penetration should not be framed as the default goal. Single patients and those without a current partner should be offered solo intimacy goals, body reconnection strategies and the option to include a trusted support person if desired.

Core nurse-led or shared actions include permission to discuss sexuality, baseline documentation of concerns and goals, explanation of the likely sexual effects of surgery, screening for pre-existing sexual dysfunction, pelvic pain, menopausal symptoms, trauma history and psychosocial risk, and provision of the diversion-specific intimacy checklist. Red flags before surgery include severe pelvic pain, complex sexual trauma history, major untreated psychiatric comorbidity, severe relationship distress, language barriers without interpreter access and limited capacity for self-care planning.

#### 2.1.6 Phase 2: early postoperative care, 0 to 8 weeks

Objectives are to protect healing, manage pain and fatigue, prevent maladaptive guarding, support safe movement, maintain dignity and normalise non-penetrative intimacy. The link between intra-abdominal pressure management and sexual rehabilitation should be made explicit: straining, poor breath control, unmanaged constipation and unsupported high-load positions may increase discomfort, appliance traction, leakage anxiety and hernia risk, all of which can reinforce avoidance of intimacy (Antoniou et al., 2018; Mills, 2023).

Early precautions include avoiding breath-holding, heavy lifting, hard straining on the toilet, high-load core work, aggressive scar massage over unhealed tissue and penetrative intercourse until surgical clearance. Early intimacy work can include non-goal-oriented touch, cuddling, sensual but non-genital contact, mirror or garment choice exercises and short communication rehearsals. Patients should be told to stop and seek review for fever, wound problems, new abdominal or stomal bulge, acute bleeding, escalating pelvic pain, uncontrolled leakage, suspected urinary diversion complication or severe psychological distress.

#### 2.1.7 Phase 3: late rehabilitation, 8 weeks and beyond

Late rehabilitation should be individually dosed and guided by healing, symptoms, goals and tolerance. For patients without partners or those choosing non-penetrative goals, all interventions can be adapted to include self-exploration, masturbation techniques and body acceptance exercises. The intervention menu is deliberately modular: not every patient needs every component. The clinician should ask what sexual recovery means to the patient, whether goals are partnered or solo, whether genital contact or penetration is desired, and what body-image or diversion-related barriers are most salient.

For erectile dysfunction, pelvic floor muscle training with breath-effort coordination, vacuum erection device education, phosphodiesterase type 5 inhibitor or intracavernosal injection referral pathways, and counselling about nerve-sparing status and realistic recovery may be considered, drawing on pelvic cancer rehabilitation reviews and cystectomy treatment studies (Nicolai et al., 2021; Moussa et al., 2021; Titta et al., 2006; Chappidi et al., 2017). Recovery counselling should also account for nerve-sparing status and reported recoverability after radical cystectomy (Hekal et al., 2009). Orthotopic neobladder patients may also need strategies for leakage or climacturia, including voiding schedules, pre-voiding, pelvic contraction timing, positioning and contingency planning (Pronk et al., 2023).

For vaginal dryness, dyspareunia, arousal changes or altered genital sensation, assessment should consider tissue healing, pelvic floor tone, scars, prolapse symptoms, menopausal status, pain mechanisms and body-image distress. First-line self-management may include lubricants, vaginal moisturisers, pacing, arousal-first strategies and non-penetrative options; the arousal-first framing is consistent with contemporary models of female sexual response (Basson, 2000). Pelvic health physiotherapists with appropriate training may provide down-training, graded exposure, manual therapy or dilator progression when indicated. Cystectomy-specific and bladder cancer literature should inform counselling about female sexual function, vaginal changes and body image (Milling et al., 2024; Zippe et al., 2004; von Deimling et al., 2022; Martin et al., 2023). Referral to gynaecology or menopause care should be made for persistent vulvovaginal symptoms, consideration of topical vaginal oestrogen or other genitourinary syndrome of menopause therapies, systemic hormone therapy decisions, undiagnosed bleeding, complex contraindications or refractory pain (Wenk et al., 2023; Louar et al., 2022; The North American Menopause Society, 2020).

Manual therapy, internal examination and dilator work should be deferred in the presence of unhealed tissue, active infection, unexplained bleeding, severe uncontrolled pain, suspected fistula or urinary diversion complication, symptomatic prolapse requiring specialist assessment, or absence of informed consent. Any intervention should be stopped if pain, distress or autonomic symptoms escalate.

For body-image and ostomy-related intimacy concerns, stoma nursing and rehabilitation clinicians can support pouch emptying routines, seal checks, cover or belt options, clothing choices, stepwise visibility decisions, position modifications and scripts for pausing intimacy if pain, leakage, pulling or anxiety occurs. Cultural, religious, gender, disability, age and relationship contexts should be actively explored rather than assumed.

Psychosexual skills can be introduced using the Bring up, Explain, Tell, Timing, Educate and Record model and the Extended Permission, Limited Information, Specific Suggestions and Intensive Therapy model (Mick et al., 2004; Taylor and Davis, 2006). Sensate-focus principles can be adapted for partnered or solo use, starting with non-demand touch and progressing only when the patient feels safe and interested (Weiner and Avery-Clark, 2014). Referral to psychology, psycho-oncology, sexology or sexual medicine is appropriate for trauma-related symptoms, severe avoidance, persistent anorgasmia with distress, complex desire discrepancy, compulsive avoidance or relationship conflict.

#### 2.1.8 Diversion-specific tailoring

Orthotopic neobladder: emphasise voiding schedules, night-time planning, leakage and climacturia education, pelvic floor timing and positions that reduce abdominal pressure or fear of leakage. Continent cutaneous reservoir: plan catheterisation timing around intimacy, abdominal support during pressure events and skin or stoma care. Ileal conduit or urostomy: focus on pouch emptying, seal security, mini-pouches or caps where locally available, belts or covers, body-image coaching and positions that avoid traction, pressure and appliance shear.

#### 2.1.9 Role mapping and referral triggers

Table 2 translates the protocol into delivery levels so that services can adapt the framework to local professional scope and staffing. Actual division of labour should be based on local training, credentialing and regulation; the table is a general implementation guide rather than a universal scope-of-practice rule.

**Table 2:** Delivery-level mapping for implementation and referral.

Component	Nurse-led or stoma nurse-led	Shared interdisciplinary	Specialist referral only
Routine sexual health conversation	Permission-giving, normalisation, privacy, documentation and written information.	Joint counselling when pelvic rehabilitation or complex diversion concerns are present.	Intensive psychosexual therapy for complex distress, trauma or relationship conflict.
Diversion-specific intimacy planning	Pouch or reservoir preparation, garment choices, leakage and odour planning, skin checks.	Position planning with physiotherapy when pain, abdominal pressure or mobility limits are present.	Review by urology or stoma specialist for recurrent leakage, skin breakdown or suspected diversion complication.
Pelvic floor and activity progression	Screening questions, safe toileting advice, referral initiation.	External pelvic coordination, graded activity, breath strategies and outcome tracking.	Internal examination, manual therapy and complex pain management only by credentialed clinicians.
Medication and devices	Identify concerns and support adherence to prescribed plans.	Rehabilitation coaching alongside prescribed treatment.	Prescribing or initiating hormone therapy, erectile dysfunction medication, injection therapy or device pathways.
Body image and distress	Screening, supportive conversation, appliance visibility choices.	Body-image coaching, graded exposure and partner-inclusive problem solving.	Psychology, psycho-oncology, sexology or sexual medicine for severe or persistent distress.
Solo-focused interventions	Permission-giving for solo goals, body acceptance language, privacy and self-exploration education.	Body reconnection, graded exposure, masturbation techniques and non-penetrative intimacy planning when within local competence.	Psychology, psycho-oncology, sexology or sexual medicine for trauma-related symptoms, severe avoidance or persistent distress.

#### 2.1.10 Practical intimacy checklist for patients with an ostomy or urinary diversion

• Empty the pouch or reservoir beforehand and check the seal.
• Consider a fabric cover, support belt, wrap or clothing option that increases comfort.
• Agree on a pause or stop signal before intimacy.
• Start with non-goal-oriented touch if anxiety, pain or body-image distress is present.
• Use side-lying or supported positions first if abdominal pressure, pulling or fatigue is a concern.
• Stop for pain, leakage, pulling, bleeding, new bulging, breathlessness or escalating anxiety.

## 3 Discussion

This revised framework integrates sexual health communication, pelvic rehabilitation, stoma-related intimacy planning and referral into a staged cystectomy pathway. The main implementation change is that the framework now separates actions that most oncology or stoma nurses can initiate from interventions requiring pelvic health physiotherapy, medical prescribing or psychosexual specialist care. This responds to international variation in nursing scope and reduces the risk that readers interpret the framework as requiring all centres to provide specialist sexual therapy.

The framework also makes feasibility more explicit. A full pathway may require several contacts over months, but a limited-resource service can begin with screening, permission-giving, written education, diversion-specific planning and referral triggers. Process indicators can help services audit whether sexual health is being addressed before outcome benchmarks are established.

Sex, gender and inclusivity require careful handling. Sex-specific sections in this framework refer to anatomy, surgery and physiological symptoms commonly associated with male or female reproductive anatomy; they should not be used to infer identity, relationship structure or goals. Clinicians should ask about anatomy, terminology, dysphoria triggers, cultural preferences, disability-related needs, solo or partnered goals and preferred support persons. Gender-diverse patients should be offered gender-affirming referral when needed.

### 3.1 Limitations

The evidence base specific to radical cystectomy remains limited and heterogeneous, especially for women, non-nerve-sparing surgery, people without partners, gender-diverse patients and diversion-specific rehabilitation. Qualitative work also indicates that relational, embodied and pain-related experiences after cystectomy may not be captured by sexual function scores alone (Lofgren et al., 2023). Several recommendations are extrapolated from pelvic oncology, ostomy, menopause, sexual medicine and cancer survivorship literature. The consensus process was author-panel based rather than a formal Delphi process with external patient, partner and nursing panels. The proposed dose and process indicators should therefore be treated as starting points for adaptation and audit, not as proven standards.

Long-term sexual recovery beyond 12 months is insufficiently described in the literature. The framework may also be difficult to implement in centres with limited pelvic health physiotherapy, stoma nursing time, psychosexual referral access or interpreter support. These constraints should be documented during implementation so that service development focuses on realistic barriers rather than individual clinician responsibility alone.

### 3.2 Research agenda

• Prospective feasibility studies testing the minimum viable pathway in routine cystectomy services.
• Patient and partner co-design studies, including single patients and people with non-partnered intimacy goals.
• Implementation studies comparing nurse-led screening plus referral with more intensive multidisciplinary rehabilitation models.
• Studies focused on women, non-nerve-sparing surgery, diversion type, gender-diverse patients and culturally diverse populations.
• Longitudinal studies assessing sexual function, body image, intimacy and service use with at least 24-month follow-up, including delayed sexual dysfunction patterns such as late-onset pelvic floor dysfunction, prolapse symptoms, pain recurrence, diversion-related intimacy avoidance and changing support needs.
• Evaluation of process indicators, outcome measures and clinically meaningful change thresholds in cystectomy survivorship.

## 4 Conclusions

A structured, phase-based and scope-sensitive approach can make sexual recovery after radical cystectomy more visible, safer and more person-centred. The revised framework is intended for cautious implementation within multidisciplinary cystectomy pathways, especially those involving oncology nursing, stoma nursing and pelvic health physiotherapy. Its next step is prospective evaluation with patient, partner and nursing co-design.

## Declaration of generative artificial intelligence and artificial intelligence-assisted technologies in the manuscript preparation process

During revision, artificial intelligence-assisted tools were used to support organisation of reviewer responses, language editing, abbreviation expansion and document formatting. Fig. 1 is a deterministic schematic prepared from the protocol content using plotting software; no generative image model was used. The authors reviewed and edited the content as needed and take full responsibility for the content of the submitted article.

## Funding

This research did not receive any specific grant from funding agencies in the public, commercial or not-for-profit sectors.

## Data availability statement

No new data were generated or analysed for this article. The framework is based on published literature and author-panel consensus.

## CRediT authorship contribution statement

**Marek Broul:** Writing – review & editing, Writing – original draft, Validation, Methodology, Investigation, Conceptualization. **Aneta Hujova:** Writing – review & editing, Methodology, Formal analysis, Data curation. **Michaela Liegertova:** Writing – review & editing, Writing – original draft, Methodology. **Jaroslava Banyrova:** Writing – review & editing, Writing – original draft, Methodology, Investigation, Formal analysis, Data curation. **Michal Vostry:** Supervision, Project administration, Methodology. **Klara Dadova:** Writing – review & editing, Supervision, Methodology.

## Declaration of competing interest

The authors declare that they have no known competing financial interests or personal relationships that could have appeared to influence the work reported in this paper.
